# Costs and Cost-Effectiveness of 9-Valent Human Papillomavirus (HPV) Vaccination in Two East African Countries

**DOI:** 10.1371/journal.pone.0106836

**Published:** 2014-09-08

**Authors:** Sorapop Kiatpongsan, Jane J. Kim

**Affiliations:** 1 Harvard Interfaculty Initiative in Health Policy, Cambridge, Massachusetts, United States of America; 2 Department of Obstetrics and Gynecology, Faculty of Medicine, Chulalongkorn University, Bangkok, Thailand; 3 Sasin Graduate Institute of Business Administration of Chulalongkorn University, Bangkok, Thailand; 4 Center for Health Decision Science, Harvard School of Public Health, Boston, Massachusetts, United States of America; Penn State University School of Medicine, United States of America

## Abstract

**Background:**

Current prophylactic vaccines against human papillomavirus (HPV) target two of the most oncogenic types, HPV-16 and -18, which contribute to roughly 70% of cervical cancers worldwide. Second-generation HPV vaccines include a 9-valent vaccine, which targets five additional oncogenic HPV types (i.e., 31, 33, 45, 52, and 58) that contribute to another 15–30% of cervical cancer cases. The objective of this study was to determine a range of vaccine costs for which the 9-valent vaccine would be cost-effective in comparison to the current vaccines in two less developed countries (i.e., Kenya and Uganda).

**Methods and Findings:**

The analysis was performed using a natural history disease simulation model of HPV and cervical cancer. The mathematical model simulates individual women from an early age and tracks health events and resource use as they transition through clinically-relevant health states over their lifetime. Epidemiological data on HPV prevalence and cancer incidence were used to adapt the model to Kenya and Uganda. Health benefit, or effectiveness, from HPV vaccination was measured in terms of life expectancy, and costs were measured in international dollars (I$). The incremental cost of the 9-valent vaccine included the added cost of the vaccine counterbalanced by costs averted from additional cancer cases prevented. All future costs and health benefits were discounted at an annual rate of 3% in the base case analysis. We conducted sensitivity analyses to investigate how infection with multiple HPV types, unidentifiable HPV types in cancer cases, and cross-protection against non-vaccine types could affect the potential cost range of the 9-valent vaccine. In the base case analysis in Kenya, we found that vaccination with the 9-valent vaccine was very cost-effective (i.e., had an incremental cost-effectiveness ratio below per-capita GDP), compared to the current vaccines provided the added cost of the 9-valent vaccine did not exceed I$9.7 per vaccinated girl. To be considered very cost-effective, the added cost per vaccinated girl could go up to I$5.2 and I$16.2 in the worst-case and best-case scenarios, respectively. At a willingness-to-pay threshold of three times per-capita GDP where the 9-valent vaccine would be considered cost-effective, the thresholds of added costs associated with the 9-valent vaccine were I$27.3, I$14.5 and I$45.3 per vaccinated girl for the base case, worst-case and best-case scenarios, respectively. In Uganda, vaccination with the 9-valent vaccine was very cost-effective when the added cost of the 9-valent vaccine did not exceed I$8.3 per vaccinated girl. To be considered very cost-effective, the added cost per vaccinated girl could go up to I$4.5 and I$13.7 in the worst-case and best-case scenarios, respectively. At a willingness-to-pay threshold of three times per-capita GDP, the thresholds of added costs associated with the 9-valent vaccine were I$23.4, I$12.6 and I$38.4 per vaccinated girl for the base case, worst-case and best-case scenarios, respectively.

**Conclusions:**

This study provides a threshold range of incremental costs associated with the 9-valent HPV vaccine that would make it a cost-effective intervention in comparison to currently available HPV vaccines in Kenya and Uganda. These prices represent a 71% and 61% increase over the price offered to the GAVI Alliance ($5 per dose) for the currently available 2- and 4-valent vaccines in Kenya and Uganda, respectively. Despite evidence of cost-effectiveness, critical challenges around affordability and feasibility of HPV vaccination and other competing needs in low-resource settings such as Kenya and Uganda remain.

## Introduction

Prophylactic vaccines against human papillomavirus (HPV) represent a major breakthrough in cancer prevention. Two available HPV vaccines target two of the most oncogenic types, HPV-16 and -18, that contribute to roughly 70% of cervical cancers – and a smaller proportion of other anogenital and oral cancers – worldwide [1]; one of these vaccines also targets non-oncogenic types, HPV-6 and -11, which cause the majority of genital warts. Second-generation HPV vaccines include a 9-valent (“nonavalent”) vaccine, which targets five additional oncogenic HPV types (i.e., 31, 33, 45, 52, and 58) that contribute to another 15–30% of cervical cancer cases [2]. Merck & Co., Inc. announced in November 2013 that its investigational 9-valent HPV vaccine (V503) prevented approximately 97% of cervical, vaginal and vulvar pre-cancers caused by HPV types 31, 33, 45, 52, and 58 in a Phase III efficacy study. The new vaccine also generated non-inferior immune responses to HPV types 6, 11, 16, and 18 compared with those generated by the current 4-valent HPV vaccine [3], [4].

In a previous model-based analysis, under similar efficacy assumptions, we demonstrated that the 9-valent vaccine yields greater reductions in cervical cancer, compared to currently-available 2- and 4-valent vaccines [5]. We explored several uncertainties with respect to HPV infections and vaccine properties and found that the prevalence of co-infection with multiple HPV types and unidentifiable HPV types in cancer cases can influence estimates of vaccine effectiveness, but that the magnitude of effect may be moderated by vaccine cross-protection against HPV types not targeted by the vaccine [5]. For countries considering adoption of HPV vaccination, information on how the added benefit of the 9-valent vaccine weighs against its cost will be critical.

This report is intended as a follow-up to the previous analysis of the population-level health effects of HPV vaccination with the 9-valent vaccine in Kenya and Uganda [5] and focuses on determining a range of vaccine costs for which the 9-valent vaccine would be cost-effective in comparison to the current HPV vaccines. As in our previous analysis [5], we explored the influences of co-infection with multiple HPV types, unidentifiable HPV types, and cross-protection against non-targeted HPV types on the cost thresholds of the 9-valent vaccine.

## Methods

**Figure 1:**
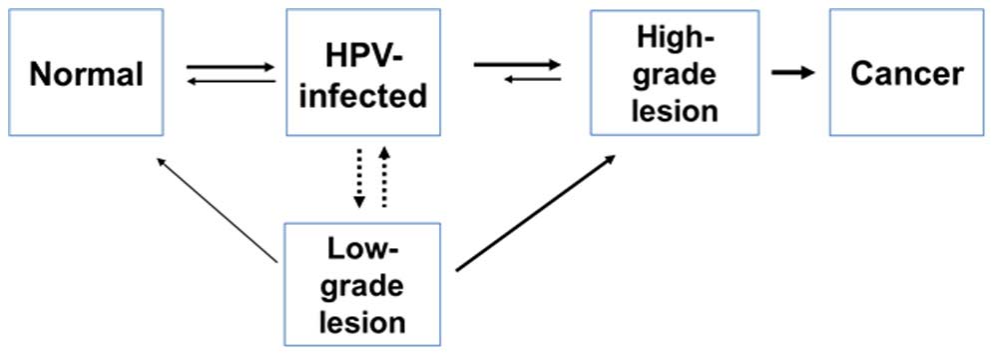
Disease simulation model of HPV and cervical cancer. The model comprises mutually-exclusive and collectively-exhaustive health states that represent key elements of HPV infection and cervical carcinogenesis. Individual women are simulated in the model from an early age (age 9) and transition through these health states over the lifetime. Health events, outcomes, and resource use are tracked and aggregated at the population level.

The analysis was performed in the context of two separate countries, Kenya and Uganda, using a natural history disease simulation model of HPV and cervical cancer. The mathematical model simulates individual women from an early age (age 9) and tracks health events and resource use as they transition through clinically-relevant health states over their lifetime (Figure 1). We performed the analysis based on a single cohort of 1,000,000 preadolescent girls in Kenya and in Uganda. The disease simulation model used in this study was a static (non-dynamic) stochastic model. Therefore, this model did not account for herd-immunity effects.

Epidemiological data on HPV prevalence and cancer incidence were used to adapt the model to each country in a calibration process that has been described in previous publications [5], [6]. In brief, the model calibration was performed in two steps. In the first step, primary epidemiologic data were used to identify a plausible range for each natural history input parameter. In the second step, a simultaneous search over all input parameters was performed to identify parameter sets that produced outputs consistent with empirical calibration target data. The calibrated model had reasonable fit to the empirical target data with respect to duration and prevalence of high-risk HPV infections, prevalence of precancerous lesions, and incidence of cervical cancer. A complete description of the model and a complete description of the model calibration process were previously reported [7]. Kenya and Uganda were selected because of the range in contribution of HPV-16 and -18 in cervical cancer cases, 62.2% and 73.9% respectively [2], as well as the absence of organized cervical cancer screening programs.

In the current analysis, we used the model to determine a potential cost range of the 9-valent vaccine that would make it cost-effective in comparison to the current HPV vaccines in Kenya and Uganda. Cost-effectiveness was expressed in terms of the incremental cost-effectiveness ratio, defined as the additional cost of the 9-valent vaccine divided by the additional health benefit, compared to the current (2- or 4-valent) vaccines. We assumed that HPV vaccination occurred in pre-adolescence (by age 12) prior to sexual debut, and coverage for all three required doses was 100% for any of the HPV vaccines. We assumed 100% vaccine efficacy against targeted HPV types over the lifetime for both currently available vaccines and the 9-valent vaccine as in previously published studies [5], [6]. Health benefit, or effectiveness, from HPV vaccination was measured in terms of life expectancy, and costs were measured in 2005 international dollars (I$) to make the analysis consistent with and comparable to a previously published study [6]. The incremental cost of the 9-valent vaccine included the added cost of the vaccine counterbalanced by costs averted from additional cancer cases prevented. Direct medical costs associated with diagnosis and treatment of cervical cancers included the facility visits, personnel, procedures (including pharmaceuticals and supplies), and cancer care (including staging of cancer severity, hospitalizations, stage-appropriate treatments). Consistent with the societal perspective, we also included the cost of patient transport and time spent traveling, waiting, and receiving care [6], [8]. All future costs and health benefits were discounted at an annual rate of 3% in the base case analysis.

There is no universally-accepted criterion that defines a threshold cost-effectiveness ratio below which a technology or intervention would be considered good value for money. We followed the Commission on Macroeconomics and Health's suggestion that interventions with a cost-effectiveness ratio less than a country's Gross Domestic Product (GDP) per capita could be considered “very cost-effective” and less than three times the GDP per capita could be considered ‘‘cost-effective” [9]. Using these two levels of GDP as proxies for societal willingness-to-pay for a year of life saved, we calculated the cost threshold of the 9-valent vaccine below which HPV vaccination with the 9-valent vaccine would be (very) cost-effective, compared to vaccination with the 2- or 4-valent vaccines. The GDP per capita in 2005 for Kenya was I$1470 and for Uganda was I$1077 [10].

Analyses were conducted under three different scenarios as described in detail in a previous study [5]. Briefly, in Scenario A, there is assumed to be no overlap between unidentifiable HPV types and multiple infections with HPV types that are targeted by the 9-valent vaccine, and therefore, the 9-valent vaccine does not offer any benefit in preventing these cases. For Scenario B, there is some overlap between unidentifiable HPV types and multiple infections with vaccine-targeted HPV types, and therefore, the 9-valent vaccine has the ability to prevent some of these cases, proportional to the prevalence of the five targeted HPV types relative to the prevalence of all non-16/18 types. For Scenario C, there is complete overlap between unidentifiable HPV types and multiple infections with vaccine-targeted HPV types, and therefore, the 9-valent vaccine offers full protective benefits in preventing these cases. Four possible levels of cross-protective effects (0%, 7.4%, 37.4% and 58.2%) against non-vaccine types based on vaccine trial data were incorporated into Scenarios A, B and C [11]. For the base case analysis, we selected scenario B, in which the 9-valent vaccine has the ability to prevent some cases with unidentifiable types and multiple infections, in conjunction with a moderate level of cross-protective effects against non-vaccine types (37.4%). One-way sensitivity analysis was performed to evaluate the effects of each source of uncertainty on the 9-valent vaccine cost threshold. Two-way sensitivity analysis and best- and worst-case scenarios were conducted to evaluate the simultaneous effects of multiple sources of uncertainties on the potential cost range. The most optimistic scenario for the incremental benefits of the 9-valent vaccine is if it can fully protect against unidentifiable HPV types and multiple infections (Scenario C), while also assuming that none of the vaccines (both the 9-valent vaccine and current vaccines) provide cross-protection against non-vaccine HPV types. The least optimistic scenario is if cases with unidentifiable types and multiple infections are not attributable to the additional types targeted by the 9-valent vaccine (Scenario A) while also assuming that all the vaccines provide high cross-protection against non-vaccine types. A sensitivity analysis on the discounting rate was performed to explore the effects of time preference on the vaccine cost threshold.

## Results

Table 1 shows the cost thresholds of the 9-valent HPV vaccine at which the incremental cost-effectiveness ratio of HPV vaccination with the 9-valent vaccine versus the 2- or 4-valent vaccine is equal to per-capita GDP and three times per-capita GDP under various scenarios. In the base case analysis in Kenya, vaccination with the 9-valent vaccine was *very cost-effective* (i.e., had an incremental cost-effectiveness ratio below per-capita GDP), compared to the current vaccines provided the added cost of the 9-valent vaccine did not exceed I$9.7 per vaccinated girl. In the worst-case scenario in which the 9-valent could not protect against any unidentifiable HPV types and multiple infections (Scenario A) with an assumption that the vaccines (both the 9-valent vaccine and current vaccines) provide high cross-protection against non-vaccine HPV types, this cost threshold decreased to I$5.2 per vaccinated girl. In the best-case scenario in which the 9-valent vaccine could fully protect against unidentifiable HPV types and multiple infections (Scenario C) in the absence of any cross-protection against non-vaccine HPV types, the added costs of the 9-valent vaccine could increase to I$16.2 per vaccinated girl. At a willingness-to-pay threshold of three times per-capita GDP, the thresholds of added costs associated with the 9-valent vaccine were I$27.3, I$14.5 and I$45.3 per vaccinated girl for the base case, worst-case and best-case scenarios, respectively.

**Table 1:** Thresholds of incremental cost (I$) per vaccinated girl associated with the 9-valent vaccine in Kenya and Uganda (discounting rate  = 3% per year)*.

	Kenya		Uganda	
	Willingness-to-pay thresholds			
	1x GDP per capita	3x GDP per capita	1x GDP per capita	3x GDP per capita
**Base case scenario** †				
	9.7	27.3	8.3	23.4
**One-way sensitivity analysis on multiple HPV infections & unidentifiable types**				
No benefit to prevent cervical cancer with unidentifiable types and multiple infections	7.4	20.7	6.5	18.4
Full benefit to prevent cervical cancer with unidentifiable types and multiple infections	10.8	30.4	9.1	25.5
**One-way sensitivity analysis on cross-protective effects against non-vaccine types**				
0% cross-protective effects	14.5	40.6	12.5	35.2
7.4% cross-protective effects	13.6	38.1	11.7	33.0
58.2% cross-protective effects	6.8	19.1	5.8	16.2
**Two-way sensitivity analysis on multiple infections & unidentifiable types and cross-protective effects**				
No benefit to prevent cervical cancer with unidentifiable types and multiple infections with 58.2% cross-protective effects	5.2	14.5	4.5	12.6
Full benefit to prevent cervical cancer with unidentifiable types and multiple infections with no cross-protective effects	16.2	45.3	13.7	38.4

* GDP  =  gross domestic product; I$  =  international dollars. Values represent the added cost of the 9-valent HPV vaccine at which the incremental cost-effectiveness ratio (compared to current 2- or 4-valent vaccines) would be equal to 1x or 3x per capita GDP in each country.

† Base case scenario  =  some benefits to prevent cervical cancer with unidentifiable types and multiple infections, defined as a function of the prevalence of the five targeted HPV types relative to the prevalence of all non-16/18 types, with 37.4% cross-protection against non-vaccine types.

In Uganda, the threshold cost of the 9-valent vaccine over the 2- or 4-valent vaccines was found to be lower than in Kenya across all scenarios. For example, in the base case analysis, the cost threshold for the 9-valent vaccine was I$8.3 and I$23.4 per vaccinated girl when the willingness-to-pay threshold was one time and three times per-capita GDP, respectively. From the worst-case to best-case scenarios, the threshold cost associated with the 9-valent vaccine ranged from I$4.5 to I$13.7 per vaccinated girl, assuming a willingness-to-pay threshold of per-capita GDP, and I$12.6 to I$38.4 per vaccinated girl, assuming a willingness-to-pay threshold of three times per-capita GDP.

Table 2 shows the impact of varying time preference on the threshold costs. With a discounting rate of 0%, the threshold cost per vaccinated girl of the 9-valent vaccine could increase from I$9.7 to I$50.4 in Kenya and from I$8.3 to I$41.3 in Uganda. With a discounting rate of 5% per year, the threshold cost per vaccinated girl of the 9-valent vaccine could decrease from I$9.7 to I$3.6 in Kenya and from I$8.3 to I$3.2 in Uganda.

**Table 2:** Impact of discount rate on thresholds of incremental cost (I$) per vaccinated girl associated with the 9-valent vaccine*.

	Kenya		Uganda	
	Annual discount rate			
	0%	5%	0%	5%
**Base case scenario** †				
	50.4	3.6	41.3	3.2
**One-way sensitivity analysis on multiple HPV infections & unidentifiable types**				
No benefit to prevent cervical cancer with unidentifiable types and multiple infections	38.3	2.7	32.4	2.5
Full benefit to prevent cervical cancer with unidentifiable types and multiple infections	55.9	4.0	44.9	3.4
**One-way sensitivity analysis on cross-protective effects against non-vaccine types**				
0% cross-protective effects	75.1	5.3	62.1	4.7
7.4% cross-protective effects	70.4	5.0	58.2	4.4
58.2% cross-protective effects	34.9	2.5	28.3	2.2
**Two-way sensitivity analysis on multiple infections & unidentifiable types and cross-protective effects**				
No benefit to prevent cervical cancer with unidentifiable types and multiple infections with 58.2% cross-protective effects	26.7	1.9	22.1	1.7
Full benefit to prevent cervical cancer with unidentifiable types and multiple infections with no cross-protective effects	83.7	6.0	67.7	5.2

* GDP  =  gross domestic product; I$  =  international dollars. Values represent the added cost of the 9-valent HPV vaccine at which the incremental cost-effectiveness ratio (compared to current 2- or 4-valent vaccines) would be equal to 1x per capita GDP in each country.

† Base case scenario  =  some benefits to prevent cervical cancer with unidentifiable types and multiple infections, defined as a function of the prevalence of the five targeted HPV types relative to the prevalence of all non-16/18 types, with 37.4% cross-protection against non-vaccine types.

## Discussion

This analysis estimates a range of added vaccine costs within which HPV vaccination with the 9-valent vaccine remains cost-effective compared to vaccination with current (2- or 4-valent) vaccines in Kenya and Uganda. Because of the added health benefits from vaccination with the 9-valent vaccine, we expected that additional costs could be incurred with the 9-valent vaccine compared to the current vaccines. We found that co-infection with multiple HPV types, unidentifiable HPV types, and cross-protection against non-vaccine types all influenced the potential cost range of the 9-valent vaccine. Results were also quite sensitive to the annual rate at which future costs and benefits were discounted since the health benefits of cancers averted are expected to be realized long after the upfront cost of vaccination is incurred. The discount rate will play an important role when comparing the HPV vaccines with other vaccines or health interventions that return more immediate health impact.

Despite the utility of our disease simulation model in projecting incremental cost thresholds of the 9-valent vaccine under complex interactions of various uncertainties, our model and analysis have limitations. First of all, our disease simulation model is not a dynamic model and therefore is not able to capture herd-immunity effects. However, with the assumption that there were perfect vaccine uptake and dosage completion, herd-immunity effects would have little or no influence on the findings. Secondly, we did not vary the levels of vaccine uptake and completion. Perfect vaccine uptake is unlikely even in developed countries; however, by assuming complete coverage, we were able to estimate the maximum incremental cost thresholds of the 9-valent vaccine under the different scenarios.

If we consider only the current 2- or 4-valent HPV vaccines in Kenya and Uganda, the cost per vaccinated girl should not exceed I$41.6 and I$62.5, respectively (compared to no HPV vaccination at all) to remain below a willingness-to-pay threshold of 1x per-capita GDP. At a higher willingness-to-pay threshold of 3x per-capita GDP, the cost per vaccinated girl with current 2- or 4-valent HPV vaccines in Kenya and Uganda (compared to no vaccination at all) should not exceed I$116.5 and I$176.0, respectively (Table S4 in File S1).

The presumed additional benefit of the 9-valent vaccine is also expected to come at a higher cost, primarily attributed to a higher price of the vaccine doses compared to current vaccines, since both generations of vaccines are expected to have the same delivery requirements and dosing schedule and will require similar resources, such as healthcare providers, vaccine storage, and supply chain facilities. We found that at a threshold willingness-to-pay of per-capita GDP, the incremental cost per vaccinated girl of the 9-valent HPV vaccine should be no more than I$9.7 in Kenya and I$8.3 in Uganda, implying an additional I$3.2 per dose and I$2.8 per dose, respectively. At a higher willingness-to-pay threshold of three times per-capita GDP, the incremental cost of the 9-valent vaccine should be no more than I$9.1 per dose in Kenya and I$7.8 per dose in Uganda, compared to current vaccines. Note that we focused our analysis on vaccine benefit to cervical cancer cases. The cost estimates do not reflect vaccine benefit to other non-cervical HPV related diseases. HPV-related non-cervical cancer cases are predominantly attributable to HPV type 16 which is already targeted by currently available vaccines [12]. As a result, we would not expect the 9-valent vaccine to yield much more additional benefit to non-cervical cancer cases.

Unprecedented low prices of $4.5 per dose for Merck's Gardasil (4-valent) vaccine and $4.6 per dose for GlaxoSmithKline's Cervarix (2-valent) vaccine have recently been negotiated through the GAVI Alliance, a public-private alliance dedicated to facilitating access to immunization programs in the poorest countries [13]. Both Kenya and Uganda are eligible to apply for GAVI Alliance support for HPV vaccination, and in May 2013, Kenya became the first country of seven sub-Saharan African countries to win support from the GAVI Alliance to initiate an HPV vaccination program for girls [14]. It is uncertain how much higher the manufacturer will price the 9-valent vaccine compared to current vaccines. Also, it is uncertain whether the GAVI Alliance will be able to successfully negotiate the price of the 9-valent vaccine as it did for the current vaccines. An additional I$3.2 per dose in Kenya and I$2.8 per dose in Uganda for the 9-valent vaccine (assuming a cost-effectiveness threshold of per-capita GDP) represent a 71% and 61% increase over the price offered to the GAVI Alliance for the currently available vaccines in Kenya and Uganda, respectively. At these prices, HPV vaccination of a single cohort of 12-year old girls with the 9-valent vaccine instead of the current vaccines at 100% coverage in Kenya (460,640 girls) and in Uganda (459,066 girls) [15] would require *additional* costs of approximately I$4.5 million in Kenya and I$3.8 million in Uganda per year.

In addition to assessments of value for money, a policy maker will need to carefully consider the financial burden and competing health needs, especially in resource-poor settings such as Kenya and Uganda. Specifically, in low-resource settings such as Kenya and Uganda, willingness-to-pay thresholds of 1x per-capita GDP and 3x per-capita GDP can be useful in guiding policy decisions whether to adopt new vaccines or other health interventions but should not be the only information considered. Policy makers should also consider the availability of other public programs (e.g., education, housing and water sanitation) and compare their costs and benefits in order to make appropriate resource allocations. Policy makers might find that other public programs are much more in need and are more cost-effective compared to a new HPV vaccination program. Importantly, the 9-valent HPV vaccine will need to be assessed for its feasibility, sustainability, acceptability, and competing needs based on country-specific contexts.

In summary, this study provides a threshold range of incremental costs associated with the 9-valent HPV vaccine that would make it a cost-effective intervention in comparison to currently available HPV vaccines in Kenya and Uganda. At a willingness-to-pay threshold of per-capita GDP, the added cost for the 9-valent vaccine should be no more than I$3.2 and I$2.8 per dose compared to current HPV vaccines in Kenya and Uganda, respectively. At a higher willingness-to-pay of three times per-capita GDP, the 9-valent vaccine should cost no more than I$9.1 and I$7.8 per dose in Kenya and Uganda, respectively. Despite evidence of cost-effectiveness, critical challenges around affordability and feasibility of HPV vaccination in settings such as Kenya and Uganda remain.

### Ethics Committee Approval

Ethics committee approval was not required for this study.

## Supporting Information

File S1 (DOCX)
