# Supplementary material for: Costs and Cost-Effectiveness of 9-Valent Human Papillomavirus (HPV) Vaccination in Two East African Countries
Source: PLoS One. 2014 Sep 8;9(9):e106836. doi: 10.1371/journal.pone.0106836 (PMC4157790; doi:10.1371/journal.pone.0106836)
Supplement: File S1 — (DOCX) [file pone.0106836.s001.docx]

**Supplemental Information S1**

Accompanying “Costs and Cost-Effectiveness of 9-valent Human Papillomavirus (HPV) Vaccination in Two East African Countries”

**Table S1. Total cost (I$) excluding vaccination cost per individual girl and life years (life expectancy) associated with each scenario in Kenya and Uganda (discounting rate = 3% per year) ***

|  |  | **Kenya** | | **Uganda** | |
| --- | --- | --- | --- | --- | --- |
|  |  | Total cost | Life years | Total cost | Life years |
| **Natural history** | |  |  |  |  |
|  | | 5.537 | 34.234 | 6.958 | 33.545 |
| **Currently available vaccines** | |  |  |  |  |
|  | 0% cross protective benefits | 2.187 | 34.255 | 1.750 | 33.592 |
|  | 7.4% cross protective benefits | 2.047 | 34.256 | 1.637 | 33.593 |
|  | 37.4% cross protective benefits | 1.452 | 34.259 | 1.115 | 33.597 |
|  | 58.2% cross protective benefits | 1.007 | 34.262 | 0.799 | 33.600 |
| **9-valent vaccine** | | | | | |
|  | Base case scenario † | 0.502 | 34.265 | 0.368 | 33.604 |
| **One-way sensitivity analysis on multiple HPV infections & unidentifiable types** | | | | | |
|  | No benefit to prevent cervical cancer with unidentifiable types and multiple infections | 0.726 | 34.264 | 0.536 | 33.603 |
|  | Full benefit to prevent cervical cancer with unidentifiable types and multiple infections | 0.382 | 34.266 | 0.300 | 33.605 |
| **One-way sensitivity analysis on cross-protective effects against non-vaccine types** | | | | | |
|  | 0% cross-protective effects | 0.756 | 34.264 | 0.554 | 33.603 |
|  | 7.4% cross-protective effects | 0.708 | 34.264 | 0.520 | 33.603 |
|  | 58.2% cross-protective effects | 0.346 | 34.266 | 0.260 | 33.605 |
| **Two-way sensitivity analysis on multiple infections & unidentifiable types and cross protective effects** | | | | | |
|  | No benefit to prevent cervical cancer with unidentifiable types and multiple infections with 58.2% cross-protective effects | 0.506 | 34.265 | 0.380 | 33.604 |
|  | Full benefit to prevent cervical cancer with unidentifiable types and multiple infections with no cross-protective effects | 0.592 | 34.265 | 0.454 | 33.604 |

* I$ = international dollars.

† Base case scenario = some benefits to prevent cervical cancer with unidentifiable types and multiple infections, defined as a function of the prevalence of the five targeted HPV types relative to the prevalence of all non-16/18 types, with 37.4% cross-protection against non-vaccine types

**Table S2. Incremental cost (I$) excluding vaccination cost and life years saved per individual girl associated with the 9-valent vaccine compared to currently available vaccines in Kenya and Uganda (discounting rate = 3% per year) ***

|  |  | **Kenya** | | **Uganda** | |
| --- | --- | --- | --- | --- | --- |
|  |  | Incremental cost | Life years saved | Incremental cost | Life years saved |
| **Base case scenario** † | |  |  |  |  |
|  |  | -0.950 | 0.006 | -0.787 | 0.007 |
| **One-way sensitivity analysis on multiple HPV infections & unidentifiable types** | | | | | |
|  | No benefit to prevent cervical cancer with unidentifiable types and multiple infections | -0.726 | 0.005 | -0.619 | 0.005 |
|  | Full benefit to prevent cervical cancer with unidentifiable types and multiple infections | -1.070 | 0.007 | -0.855 | 0.008 |
| **One-way sensitivity analysis on cross-protective effects against non-vaccine types** | | | | | |
|  | 0% cross-protective effects | -1.431 | 0.009 | -1.196 | 0.011 |
|  | 7.4% cross-protective effects | -1.339 | 0.008 | -1.117 | 0.010 |
|  | 58.2% cross-protective effects | -0.661 | 0.004 | -0.539 | 0.005 |
| **Two-way sensitivity analysis on multiple infections & unidentifiable types and cross-protective effects** | | | | | |
|  | No benefit to prevent cervical cancer with unidentifiable types and multiple infections with 58.2% cross-protective effects | -0.501 | 0.003 | -0.419 | 0.004 |
|  | Full benefit to prevent cervical cancer with unidentifiable types and multiple infections with no cross-protective effects | -1.595 | 0.010 | -1.296 | 0.011 |

* I$ = international dollars.

† Base case scenario = some benefits to prevent cervical cancer with unidentifiable types and multiple infections, defined as a function of the prevalence of the five targeted HPV types relative to the prevalence of all non-16/18 types, with 37.4% cross-protection against non-vaccine types.

**Table S3. Incremental cost (I$) excluding vaccination cost and life years saved per individual girl associated with currently available vaccines compared to no vaccination in Kenya and Uganda (discounting rate = 3% per year) ***

|  |  | **Kenya** | | **Uganda** | |
| --- | --- | --- | --- | --- | --- |
|  |  | Incremental cost | Life years saved | Incremental cost | Life years saved |
| **Base case scenario** | |  |  |  |  |
|  | 37.4% cross-protective effects | -4.085 | 0.025 | -5.803 | 0.053 |
| **One-way sensitivity analysis on cross-protective effects against non-vaccine types** | | | | | |
|  | 0% cross-protective effects | -3.350 | 0.021 | -5.208 | 0.047 |
|  | 7.4% cross-protective effects | -3.491 | 0.022 | -5.321 | 0.048 |
|  | 58.2% cross-protective effects | -4.530 | 0.028 | -6.160 | 0.056 |

* I$ = international dollars.

**Table S4. Thresholds of incremental cost (I$) per vaccinated girl associated with current 2-valent or 4-valent vaccines compared to no vaccination in Kenya and Uganda (discounting rate = 3% per year) ***

|  |  | **Kenya** | | **Uganda** | |
| --- | --- | --- | --- | --- | --- |
|  |  | Willingness-to-pay thresholds | | | |
|  |  | 1x GDP per capita | 3x GDP per capita | 1x GDP per capita | 3x GDP per capita |
| **Base case scenario** | |  |  |  |  |
|  | 37.4% cross-protective effects | 41.6 | 116.5 | 62.5 | 176.0 |
| **One-way sensitivity analysis on cross-protective effects against non-vaccine types** | | | | | |
|  | 0% cross-protective effects | 34.1 | 95.7 | 56.3 | 158.6 |
|  | 7.4% cross-protective effects | 35.5 | 99.7 | 57.5 | 161.9 |
|  | 58.2% cross-protective effects | 46.1 | 129.2 | 66.3 | 186.6 |

* GDP = gross domestic product; I$ = international dollars. Values represent the added cost of current 2-valent or 4-valent HPV vaccines at which the incremental cost-effectiveness ratio (compared to no vaccination) would be equal to 1x or 3x per capita GDP in each country.
